# Pennsylvania State Veterinary Medical Association

**Published:** 1901-07

**Authors:** 


### SOCIETY PROCEEDINGS.

### PENNSYLVANIA STATE VETERINARY MEDICAL ASSOCIATION.

THE following report of Corresponding Secretary for the year ending March 5 and 6, 1901, was omitted from its proper place (May JOURNAL, page 339).

In making the first report as Corresponding Secretary of this Association for the past year it gives me much pleasure to bring before you the following:

*1st.* The great support of each individual member of this body, and the kind communications from them during the past year.

*2d.* The untiring assistance of the fellow officers and especially of our worthy President, Dr. Harger.

*3d.* The general interest manifested in the association, and I can truthfully say I know of no dissension among any of the members, and I am safe to say that it is largely due to this fact that matters have progressed so rapidly and successfully.

*4th.* The general recognition of the character and standing of this Association as a body, as well as individually, for the other State associations must naturally look to the Keystone State for the best.

*5th.* The unlimited assistance from the State Live-stock Sanitary Board under the direction of its worthy Secretary.

*6th.* The able responses to calls for papers; and right here let me say it is a great pleasure to the Secretary of any association to have a large programme and one which is represented by men from all parts of the State.

*7th.* The kindly co-operation of the journals, especially the JOURNAL OF COMPARATIVE MEDICINE AND VETERINARY ARCHIVES.

*8th.* The largely attended meeting (and profitable as well) at Erie, and the entertainment by the local committee of arrangements under the chairmanship of Dr. J. B. Irons. At this meeting we had the pleasure of electing seven men who take great interest in the Association from the fact of their being here now.

*9th.* The able and exhaustive reports of our several committees as well as the County Secretaries. In general, I think we have a great many things to be thankful for, and to make next year better than the past let us *all* do our share to make this the best State Association of its kind in the country.

| | |
|---|---|
| Up to date I have received as dues . . . | $82 00 |
| Expended . . . . . . . . . . | 67.00 |
| Balance turned over to Treasurer (March 4, 1901) . | $14.81 |
| Number of active members . . . . . . | 142 |
| Corresponding members . . . . . . . | 4 |
| Deceased . . . . . . . . . . . | 1 |
| Applications for membership . . . . . | 5 |
| Application for resignation . . . . . | 1 |

E. M. RANCK,

Secretary.

PROCEEDINGS.

(Concluded from page 405.)

Dr. Huidekoper: It is a question you must think over twice to realize the importance of. I remember the years we went through here in getting our legislation, and year after year this same thing of special legislation came up to let someone in, whose education was sadly neglected. Certainly the education, the information in regard to the law regulating veterinary medicine has been so furnished to the public through correspondence, through publications of the Association, through journals, and through public newspapers that it is absolutely incomprehensible that any man can be ignorant of the law, and of its provisions made at the time it was formed; if he does not know of it, it was that he must have been so ignorant that he could not have read anything that was published in print, or that he was not eligible to register and his wanting to do so now is an afterthought. It is now how many years? (addressing the President).

The President: Twelve years—1889.

Dr. Huidekoper (continuing): I have been out of Pennsylvania and have lost track of some of the detailed matters. With these twelve years for a precedent, and with the interest which the whole Association has taken, you have now gotten the profession on a perfectly firm basis for the future. I think that you want to combat this bill by the efforts of every individual, and, further, that you should carry it to some of the heads of the State Government. I am sure that the two United States Senators, if they were reached, would give their aid. Both Senators Quay and Penrose are deeply interested in matters affecting the veterinary public, and I am satisfied that if you go to them they will be glad to aid you. If this law should pass it would be simply setting back the profession where it was before, and open up a doorway for great evil, inviting a repetition of it year after year.

Dr. Jobson: I move that we reconsider the vote on Dr. Kooker's motion in regard to providing the Examining Board of the State with $200. You understand, gentlemen, I do not touch the question, but merely that we reconsider the vote.

The President: You all have heard the proposition made yesterday—the motion which was not carried—that this Association appropriate $200 for the use of the State Veterinary Examining Board, $200 or any part thereof to be used at their own discretion. That was the motion made yesterday morning. A Member: I second the motion.

A Member: It was to be used for the prosecution of illegal practitioners in the State.

The President: As much as they needed.

Dr. Jobson: The motion was to reopen the discussion.

The President: I simply wanted to state the proposition of the motion correctly. All those who are in favor will signify the same by saying "aye;" contrary minded, "no." The ayes have it; carried.

The question is open. Any remarks on that question? If not, a motion is in order.

The President: Does anyone wish to make a motion to appropriate $200 or part thereof?

Dr. Sallade: As President of this Board, I did not feel yesterday, when the qustion was under discussion, to place our Board in the attitude of coming before the State Association in a begging way. You are all aware that the veterinary board is an offspring from the State Association, and that the work that we perform is in the interest of the profession. You have no idea, perhaps, of the amount of work attached to this Board. The numerous complaints seem well founded, and others, perhaps, a mere endeavor on the part of the practitioner to clear his field for personal benefit to himself, and when the cases are investigated in a great many instances we find there is no ground for prosecution; in many instances we find that we succeed well in scaring off whole regiments of these illegal practitioners who either stop practising and qualify themselves, or else leave the State or desist entirely from practice. Now, we have some prosecutions, and yesterday I, as clearly and fully as I was able to do, told you how a certain case was carried on through our local court, taken up into the Superior Court, and I told you how it terminated. All those things cost money. Without money this board is absolutely tied. Last year the receipts of the board amounted to $100. We are obliged to keep an office open. Every little transaction that has been done by the board ever since its organization in 1895 is a matter of record open to inspection to you all. Now, as I said before, without money we cannot accomplish anything. The members of the board have largely contributed both of their time and their means to further the work of the board, and this matter is a matter that concerns everyone, as I said before. I think it is beneath our board to come in an humble way or in a begging way to this convention. I think that our lay boards, that the time we devote to this matter, and the money that we have expended in the furtherance of its interest should be a guarantee in itself. Now, yesterday I endeavored to refute, in as mild a manner as I possibly could, the insinuations, or a change that was made by a member of this Association, to the effect that we were careless as a board in securing evidence to convict; in other words, that we were reckless in the preparation of our cases. That is not the case. We have, as I said before, met with many difficulties. We are surrounded by difficulties all the time, and so far as I am concerned—speaking for myself individually as a member of the Board—I will say now that I am not here to beg. If you think that our efforts are a benefit to you; if you think it is necessary to carry on this Board, and you wish to have it carried on in the future, we can accomplish only so much as the means at our command will sustain.

The President: Any other remarks on that question?

A Member: I am convinced the Board has not expended this money unnecessarily, and I am in favor of giving them what they want, inside of $200.

Dr. McNeil: Being also a member of the Board, I would say, as Dr. Sallade has said, that this Board is a child of this Association, and I do not think that a member of the Board would spend one cent foolishly. The receipts for the last year, after covering the expenses, were less than $50 for the prosecutions that have been carried on and that are now being carried on. I think there are several cases that are being held under advisement, and a number of lawyers' fees to be paid. As I understood at a meeting held last night, the intention was to spend a considerable sum of money that might be appropriated by this Association in the defence of this bill introduced by Representative Shaub, and which the members of this Association are going to defend. They will defend it when reported out of committee. I think that it is the intention to spend some of that money in that way.

The President: It was not specifically so stated. Dr. McNeil (continuing): I think that is the intention of the Board. I am speaking in reference to what was talked over last night at a meeting of the majority of the Board. As a member of the Board I can say that there is very little in being a member of that Board. You get scarcely your expenses, and if some of the members did not travel on passes occasionally there would not be enough to pay the railroad fares of that Board. Then, again, as Dr. Sallade, has said, the members are constantly being roasted for neglecting to prosecute cases all over the State, whereas we have not got the funds to do it, and I think it would be right to have an appropriation.

Dr. Ranck: When I brought this matter up yesterday at the meeting, under the head of new business, it was not my intention to have as much as $200 appropriated, but I think as long as we have money in the treasury it might be spent in this way as well as any other way. It was suggested to me to bring it before the Association. Dr. Bridge said we had $322.50 in the treasury. I received yesterday $127 for dues—I have not counted up those received to-day—so that with $127 and $322 we have nearly $500 in the treasury, and if we appropriate $200 we would still have money in the treasury.

Dr. Helmer: I would say that the only trouble with the matter is that the amount is not large enough. There is not a veterinarian here but what has thought of and realized the great good that results to them from the work of this Board. This money is not a loss, but really an investment, because the work of this Board is of such a nature that it protects and elevates the profession and brings more money in your pockets. The only trouble is there is not enough; the sum is entirely too small. In my judgment there could be no better use made of the money than in that way.

Dr. Irons: I second the motion made yesterday for $200. I think it is plenty small enough for the Board to use. We certainly will expect them to use it the same as we would ourselves, for the protection of every veterinarian in the State, and I shall vote for them to have the money to-day as I voted yesterday.

Dr. Jobson: As I stated yesterday, my main objection to the passage of this motion is this: the time is inopportune for spending money on this object. If the provision had been inserted that the money was payable to the Veterinary Board, contingent upon the passage of the proposed bill, there would be no objection as far as I am concerned.

Dr. Rhodes: Mr. President: I think that I am not egotistical when I say that there are quite a number of men in the Association interested in the labor entailed in getting that little nest-egg of $200. Dr. Ranck is very quick in saying there is $500 in the treasury, but he does not count these figures as he would in his private account. There is an enormous amount of labor entailed in collecting this amount of money called for by the State Veterinary Association. We are to-day as a veterinary association—a leader—and while no doubt this Board is a child of the State Association, I think it is poor financiering to give all to one child, to the detriment of the others. There is not a man in the State Association who has a more kindly feeling for that Board than I have. I know they have not squandered a dollar. We see now that their intention is that they would like to have the money for the help of legislation now staring us detrimentally in the face at Harrisburg. I think that every veterinarian should contribute $10 to the State Board. I certainly, Mr. President, trust that these men will not vote in any other way than they did yesterday. I trust that they will not, on the spur of the moment, take this amount from the Association, for, as Dr. Helmer says, it is too small to be of any value. I say, first, we want to get legislation. They have tried case and case again; they have not a case on record that was beneficial to them. Those men whom they are fighting individually are in a position to fight them, simply from their political pull, and you know, as veterinarians, with $200 the Board can do no fighting against local politicians.

Dr. Porter: I am of the same mind as Dr. Jobson, to oppose this motion, for it is in the face of a pending resolution. It is simply foolishness; there will not be a chance for prosecution in two years. It will be now until January 1, 1902, to register. This is in relation to the prosecution of illegal practitioners. If used for any other purpose it will be illegal.

Dr. Helmer: I call for the question before the house. I move, Mr. President, that we donate or give into the hands of the State Board of Examiners the sum of $200 to be used at their discretion.

The President: At their discretion unqualifiedly? Dr. Helmer: Yes, sir.

The President: All those in favor of sustaining that motion will please give the usual sign by saying "aye," those opposing "no." The "ayes" will please rise. This is the appropriation of $200 for the prosecution of illegal registration. The motion is carried—Ayes, 21; noes, 18.

Dr. Jobson: I challenge the vote. I move you call the roll. (Motion seconded.)

Dr. Ranck (after roll-call): Gentlemen, I find twenty men vote "no" and twenty-four vote "yes."

Dr. McNeil: I wish to place on nomination the city of Pittsburgh for our next meeting-place. It has been three years since we had a meeting in Pittsburgh, and I think we had one of the best semi-annual meetings that the Association ever had, and in behalf of the veterinarians in the neighborhood of Pittsburgh I would like to invite the Association there.

A Member: I second the nomination.

Dr. Porter: I would like to name Newcastle. Dr. McNeil: I move the nomination be closed. The motion being duly seconded it was carried, and Pittsburgh decided upon.

The President: The Secretary will read the names of the new officers. (Names read by Secretary. See page 398.)

Dr. Rhodes: I, as former Secretary of this Association, want to go on record in the minutes as being thoroughly opposed to the appropriation made by this Association, which I consider is treachery to the men who are not here now, but who were here yesterday, and I want to go on record against that.

Adjourned at 6.26 P.M., *sine die*. J. C. MARSHALL, Secretary.

---

